# Protocol for reversible induction of hyposmia in rats using intranasal gadolinium chloride administration

**DOI:** 10.1016/j.xpro.2026.104943

**Published:** 2026-11-09

**Authors:** Wiktoria Podolecka, Mark Jeremy Hunt

**Affiliations:** 1 Nencki Institute of Experimental Biology of Polish Academy of Sciences, 3 Pasteur Street, 02-093 Warsaw, Poland

**Keywords:** Neuroscience, Cognitive Neuroscience, Behavior, Systems biology

## Abstract

Here, we present a protocol for intranasal administration of gadolinium chloride in adult rats to induce transient olfactory dysfunction. We describe steps for preparing gadolinium chloride solution, bilateral intranasal infusion under isoflurane anesthesia, and post-infusion stabilization. We then detail procedures for animal monitoring and behavioral assessment using the hidden cookie test. The procedure produces reproducible impairment in olfactory-guided food-seeking behavior, detectable the day after administration and lasting 7 days. Behavioral performance then returns to baseline, indicating recovery of olfactory-guided function.

## Highlights

• Instruction for training rats in the hidden cookie task to establish baseline performance
• Guidance for infusing gadolinium chloride bilaterally under isoflurane anesthesia
• Steps for maintaining rats supine for 15 min to improve solution retention
• Procedures for tracking transient hyposmia and recovery using hidden cookie testing

## Abstract (editor highlights)

Publisher’s note: Undertaking any experimental protocol requires adherence to local institutional guidelines for laboratory safety and ethics.

## Graphical abstract

**Figure:**
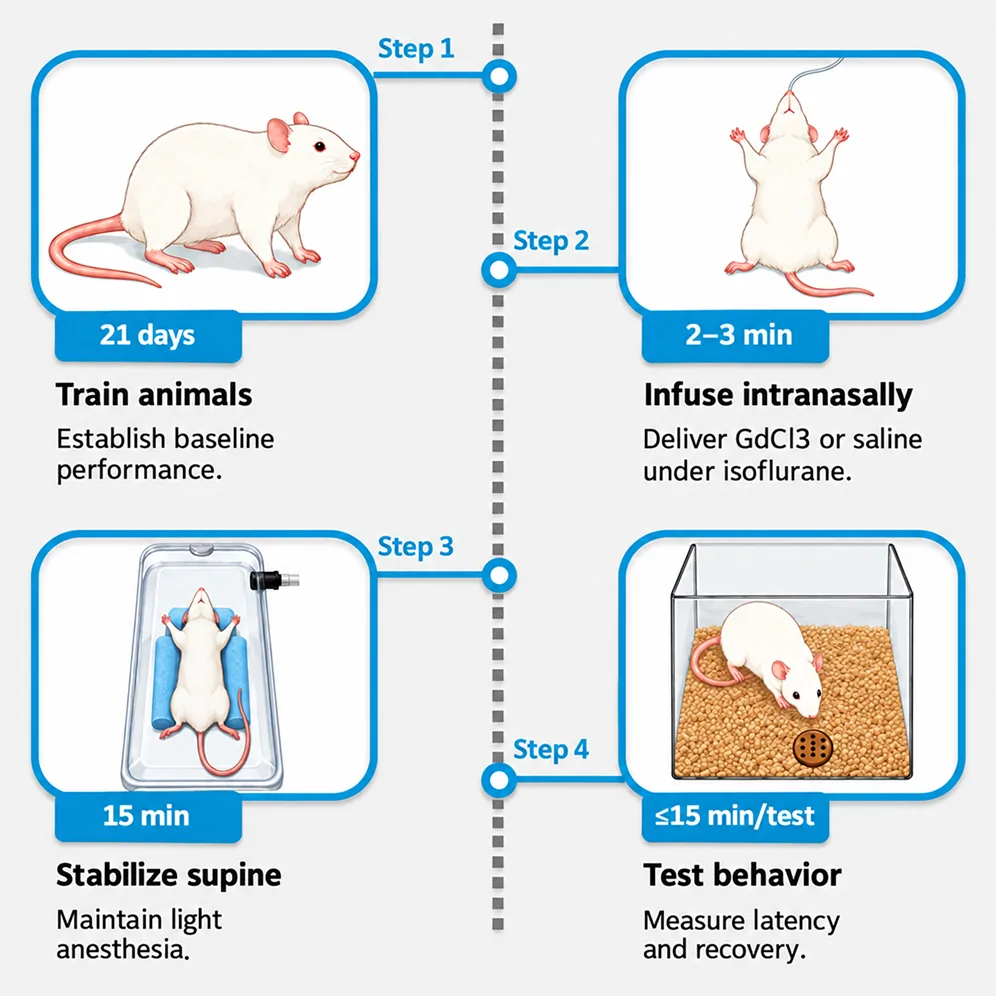

## Before you begin

Olfactory dysfunction, including hyposmia and anosmia, is a clinically relevant sensory impairment that affects food enjoyment, detection of environmental hazards, social interaction, and quality of life. Anosmia and hyposmia are estimated to affect approximately 3%–20% of the population, with risk increasing with age and in association with chronic sinonasal disease, upper respiratory tract infection, head trauma, and neurodegenerative disorders.^1^^,^^2^ In rodents, olfactory cues are particularly important for food-seeking, environmental investigation, and social communication, making experimental olfactory impairment models useful for studying the behavioral and physiological consequences of reduced smell.^3^ A reversible model is especially valuable because it allows the onset, peak, and recovery of olfactory dysfunction to be assessed within the same animals over time, without permanent ablation of the olfactory system. This protocol uses intranasal gadolinium chloride administration to induce transient hyposmia-like olfactory impairment in rats.

Because the main behavioral readout depends on olfactory-guided food-seeking, animals must first be trained to perform the hidden cookie task reliably before olfactory impairment is induced. Before intranasal gadolinium chloride or vehicle administration, habituate and train animals for 3 weeks to locate a food reward (e.g., Chocapic™ chocolate-flavored cereal) buried under 3 cm of sawdust bedding in a standard cage resembling the home cage environment (59.5 × 38 × 20 cm). This pre-training establishes comparable baseline performance across animals and ensures that post-infusion increases in retrieval latency reflect treatment-induced olfactory impairment rather than unfamiliarity with the task, the reward, or the testing environment. See “Training (before nasal infusion)” for the full training procedure.

### Innovation

This protocol provides a refined, non-surgical approach for inducing transient hyposmia-like olfactory impairment in adult rats using bilateral intranasal gadolinium chloride administration. Our recently published study showed that intranasal gadolinium produces a transient, reversible hyposmia lasting approximately 1 week, accompanied by disruption of the nasal olfactory epithelium and reduced respiration-linked oscillatory activity in the olfactory bulb.^4^ These effects were most pronounced during wakefulness, while sleep-related activity was largely preserved, providing a useful model for investigating how reduced peripheral olfactory input affects brain network activity and behavior. Compared with commonly used intranasal zinc sulfate models, which can produce variable lesion severity and may be associated with respiratory complications or mortality depending on dosing and delivery conditions, this protocol uses intranasal gadolinium chloride with optimized administration parameters to induce a robust but transient hyposmia-like phenotype.^5^^,^^6^^,^^7^ Key refinements include a defined dose and volume, careful monitoring of anesthetic depth and respiration, and a 15-min supine post-infusion stabilization period, which together improve solution retention and produce consistent impairment in the hidden cookie test followed by recovery to baseline.

The protocol also incorporates behavioral validation using a standardized hidden cookie test, pre-infusion training to establish stable baseline performance, blinded assessment, vehicle controls, repeated testing in the same animals, and welfare-focused monitoring. Hyposmia-like impairment typically emerges within 24 h, peaks during days 1–5, and resolves behaviorally within 7–10 days. Together, these features provide a practical framework for studying transient peripheral olfactory disruption and recovery without permanent olfactory ablation.

### Institutional permissions

All experimental procedures involving animals were approved by the Local Ethical Committee for Animal Experimentation at the Nencki Institute of Experimental Biology, Polish Academy of Sciences, and were conducted in accordance with European Union Directive 2010/63/EU on the protection of animals used for scientific purposes.

### Animals

Twenty 12-week-old male Wistar rats (320–400 g) were used in this protocol. Animals were obtained from the breeding facility of the Nencki Institute of Experimental Biology, Poland. The protocol was initially developed, optimized, and validated in adult male rats. Female rats were not included at this stage because additional validation is required to determine whether sex-related differences affect the optimal dose, safety profile, severity and duration of nasal epithelial damage, and recovery from hyposmia.

Rats were housed under standard environmental conditions (20–22°C, 50%–65% humidity) under a 12 h light/dark cycle (lights on at 07:00) with *ad libitum* access to food and water, except during behavioral testing.

**CRITICAL:** During pilot studies, two rats weighing <300 g died during the bilateral intranasal infusion procedure. These deaths were considered potentially related to the smaller body size of the animals and the depth of isoflurane anesthesia, which may have increased the risk of respiratory complications during the procedure. We therefore recommend using rats weighing ≥320 g and maintaining an appropriate, lighter plane of isoflurane anesthesia to minimize the risk of respiratory complications.

### Animal welfare and humane endpoints

Animals should be monitored continuously during anesthesia and until full recovery of the righting reflex. After recovery, animals should be checked at least daily for body weight, respiration, grooming, activity, posture, food and water intake, nasal discharge, and signs of distress (e.g., including lethargy, hunched posture, piloerection, reduced grooming). Animals showing persistent respiratory distress, cyanosis, severe lethargy, inability to access food or water, or body weight loss exceeding the locally approved humane endpoint (e.g., a loss of ≥20% of baseline body weight) should be removed from the study and evaluated by veterinary staff.

## Key resources table

**Table:** 

REAGENT or RESOURCE	SOURCE	IDENTIFIER
**Chemicals, peptides, and recombinant proteins**		

Gadolinium (III) chloride hydrate	Sigma Aldrich	CAS# 19423–81–5
0.9% sterile saline (NaCl solution)	Polpharma	EAN: 5903060603394
Isoflurane	Virbac	N/A
Vaseline	Vaseline	N/A

**Experimental models**		

Rattus norvegicus (Wistar) (male over 12 weeks >320 g)	Nencki Institute breeding facility	N/A

**Other**		

Isoflurane anesthesia vaporizer	RWD	R5835
Veterinary Oxygen Concentrator	RWD	ROC-5A
Induction chamber	RWD	V101-V
Fluosorber Activated Charcoal Filter Canister	Harvard Apparatus	34–0415
Silicone tubing (flexible, 0.03 inch internal diameter, 0.065 inch outer diameter, 20 cm length)	Dow Corning	602–175
Micropipette (10–100 μL range)	Discovery Comfort	4054-D
Standard rat cage (59.5 × 38 × 20 cm)	N/A	N/A
Bedding (sawdust)	N/A	N/A
Digital stopwatch	N/A	N/A
Chocapic® chocolate-flavored cereal	Nestlé	N/A

## Step-by-step method details

### Preparation for nasal infusion

**Timing: 5–10 min**

This section describes preparation of the gadolinium chloride solution and induction of anesthesia before intranasal infusion. Prepare the solution fresh on the day of use and proceed only when a stable anesthetic depth has been achieved. The equipment used for intranasal infusion is shown in Figure 1.

1. Prepare gadolinium chloride solution.
  a. Dissolve 60 mg gadolinium chloride (Gd(III) chloride hydrate ≥99.9% trace metals basis) in 1 mL sterile 0.9% saline.
  b. Mix until fully dissolved. This yields a gadolinium chloride concentration of 60 mg/mL. Each animal receives 50 μL per naris, corresponding to a total volume of 100 μL and a total dose of 6 mg gadolinium chloride per animal.
  c. Load 50 μL of solution into a 10–100 μL pipette fitted with flexible silicone tubing (0.03 inch internal diameter, 0.065 inch outer diameter, 20 cm length).

***Note:*** Prepare fresh solution on the day of use. Do not store prepared solution.

***Note:*** Gadolinium chloride should be handled using appropriate personal protective equipment and disposed of according to institutional chemical safety procedures.

2. Prepare the rat.
  a. Record animal ID, body weight, sex, genotype, operator name, and procedure date.
  b. Place the rat into an induction chamber supplied with 5% isoflurane in oxygen at 1.5–2.0 L/min.
  c. Monitor animals continuously during induction.
  d. After 3–4 min, assess anesthetic depth using toe pinch reflex and whisker response.
  e. Proceed only when an appropriate anesthetic depth is achieved, defined as absence of toe pinch reflex, absence of whisker movement, and regular respiration (40–50 breaths/min).

**CRITICAL:** Maintain anesthesia for an additional 1 min after loss of reflexes to ensure stable anesthetic depth.

**Figure 1:**
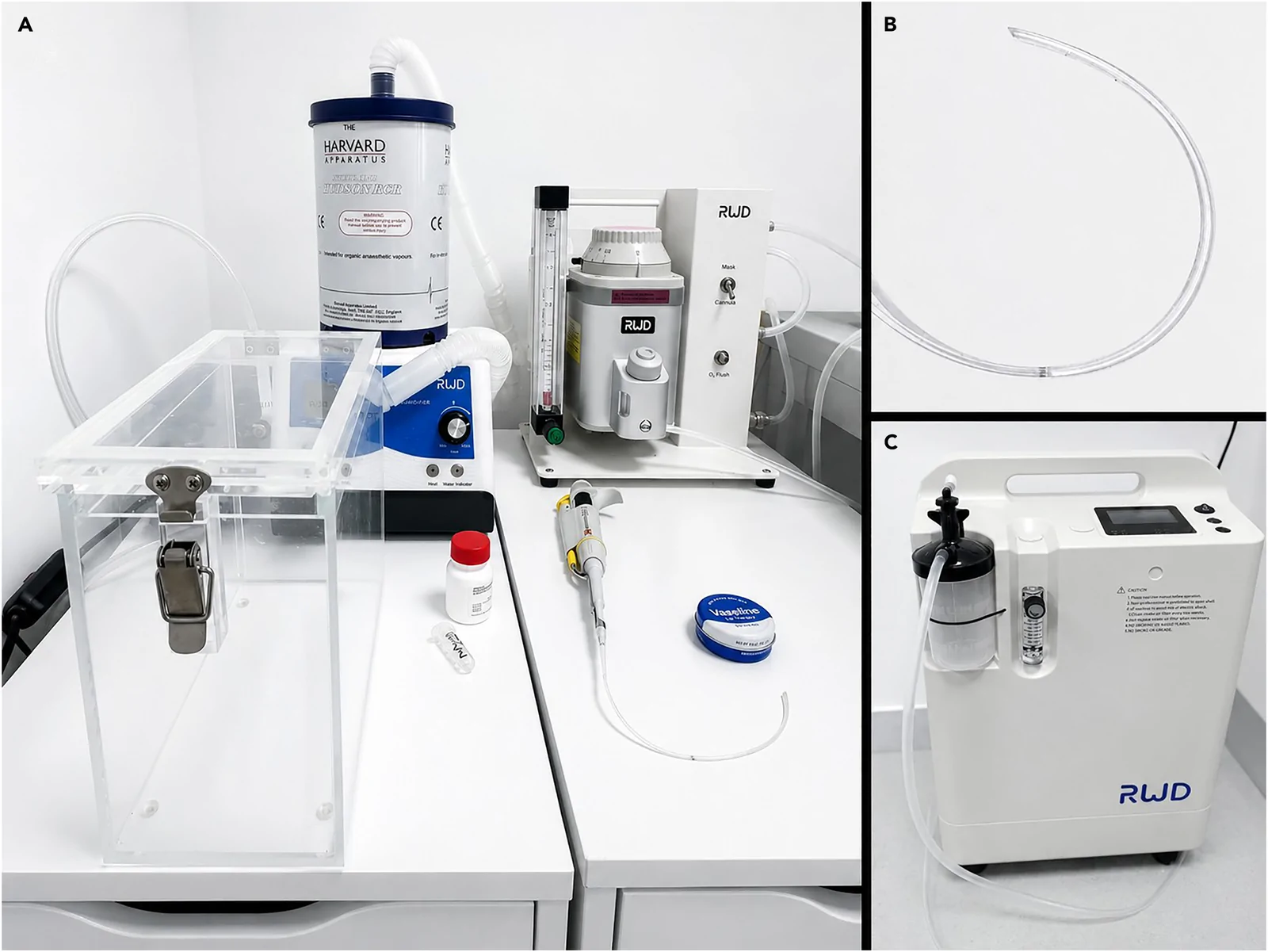
Equipment used for intranasal infusion (A) Isoflurane anesthesia chamber used for induction before intranasal infusion and for maintenance of light anesthesia with the rat positioned supine after infusion. (B) Flexible tubing with a slightly tapered tip to facilitate insertion into the naris. The black dot marks the level corresponding to 50 μL and serves as a guide to standardize the delivered volume across animals. (C) Oxygen generator.

### Intranasal gadolinium injection

**Timing: 2–3 min**

This section describes bilateral intranasal delivery of gadolinium chloride or vehicle under isoflurane anesthesia. Careful positioning, gentle tubing insertion, and continuous monitoring of respiration are essential to ensure consistent delivery while minimizing epithelial injury, solution loss, and respiratory compromise.

3. Position the rat for intranasal infusion.
  a. Remove the rat from the isoflurane induction chamber and place it in a dorsal supine position on a flat surface.
  b. Apply a thin, even coating of Vaseline to the external nares and to the tip of the flexible tubing to provide lubrication and facilitate smooth tubing insertion.
  c. Elevate the head approximately 20–30° using a rolled towel or support.

**CRITICAL:** Ensure that Vaseline does not enter or obstruct the tubing lumen, as this may prevent accurate delivery of gadolinium chloride solution.

4. Deliver gadolinium chloride or vehicle intranasally.
  a. Insert the flexible silicone tubing into the external naris to a depth of approximately 5–7 mm, or until mild resistance is encountered at the posterior nasal passage.
  b. During insertion, gently rotate the tubing while advancing it rather than applying force.
    **CRITICAL:** Do not force insertion beyond resistance to avoid epithelial injury or misplacement.
  c. Deliver 50 μL gadolinium chloride solution into one naris as a bolus.
  d. Ensure uninterrupted spontaneous respiration during delivery, defined as continuous chest wall movement without apnea lasting longer than 5 s.
  e. After the full 50 μL volume has been delivered, keep the tubing in place briefly to allow the solution to clear from the tubing tip, then slowly withdraw the tubing from the naris. Avoid rapid removal, as this may cause residual fluid to spill from the naris.
  f. Repeat the tubing insertion, bolus delivery, respiration monitoring, and tubing withdrawal steps in the contralateral naris.
  g. For vehicle-control animals, administer 50 μL sterile 0.9% saline per naris using the same route, anesthesia conditions, post-infusion stabilization period, and behavioral testing schedule as for gadolinium-treated animals.
    **CRITICAL:** Gadolinium chloride-treated animals may recover reflexes more rapidly than saline-treated animals during administration. Complete each intranasal delivery efficiently while maintaining appropriate anesthetic depth to minimize solution loss due to sneezing or snorting.
    **CRITICAL:** If the rat shows any signs of recovery during the procedure, such as whisker movement, return of reflexes, or sensitivity to nasal insertion, return the animal to the induction chamber until an appropriate anesthetic depth is re-established.
    **CRITICAL:** For inexperienced operators, it may be easier to administer the solution to one naris at a time, re-inducing anesthesia between sides if necessary. If administering one naris at a time, return the rat to the induction chamber in a supine position for 2–3 min, or until an appropriate anesthetic depth is re-established, before administering the solution to the contralateral naris. When returning the animal to the induction chamber between intranasal infusions, keep the rat’s head in a neutral, flat position rather than rotated to one side to reduce solution loss and minimize airway compromise.

### Post-infusion stabilization

**Timing: 15 min**

This section describes the post-infusion stabilization period, which is critical for reducing loss of intranasally administered solution and improving reproducibility of olfactory impairment. Animals should remain under light isoflurane anesthesia in a supine position while respiration and recovery are continuously monitored (Figure 2).

5. Maintain the rat in a supine position under light anesthesia.
  a. Immediately following bilateral infusion, move the animal in a supine position back to the induction chamber and maintain under light isoflurane anesthesia for 15 min (1%–1.5% isoflurane, oxygen flow 0.2 L/min).
  b. Ensure anesthesia depth is sufficient to prevent the righting reflex while maintaining spontaneous respiration. Isoflurane concentration and oxygen flow may need to be adjusted for individual animals.
6. Monitor recovery after stabilization.
  a. After the 15-min stabilization period, discontinue isoflurane and place the animal in a warmed recovery cage in a prone position.
  b. Monitor until the righting reflex and normal spontaneous movement return.
  c. Return the animal to the home cage only after full recovery from anesthesia.

***Note:*** Rats treated with intranasal gadolinium chloride may display an initial period of hyperactivity followed by reduced activity or withdrawal lasting 2–3 h.

**CRITICAL:** The animal must remain in a supine position for the full 15-min stabilization period. Premature recovery increases inter-animal variability due to loss of intranasal solution.

**CRITICAL:** Continuously monitor respiratory function. If respiratory distress occurs, discontinue anesthesia and reposition the animal to restore airway patency.

**Figure 2:**
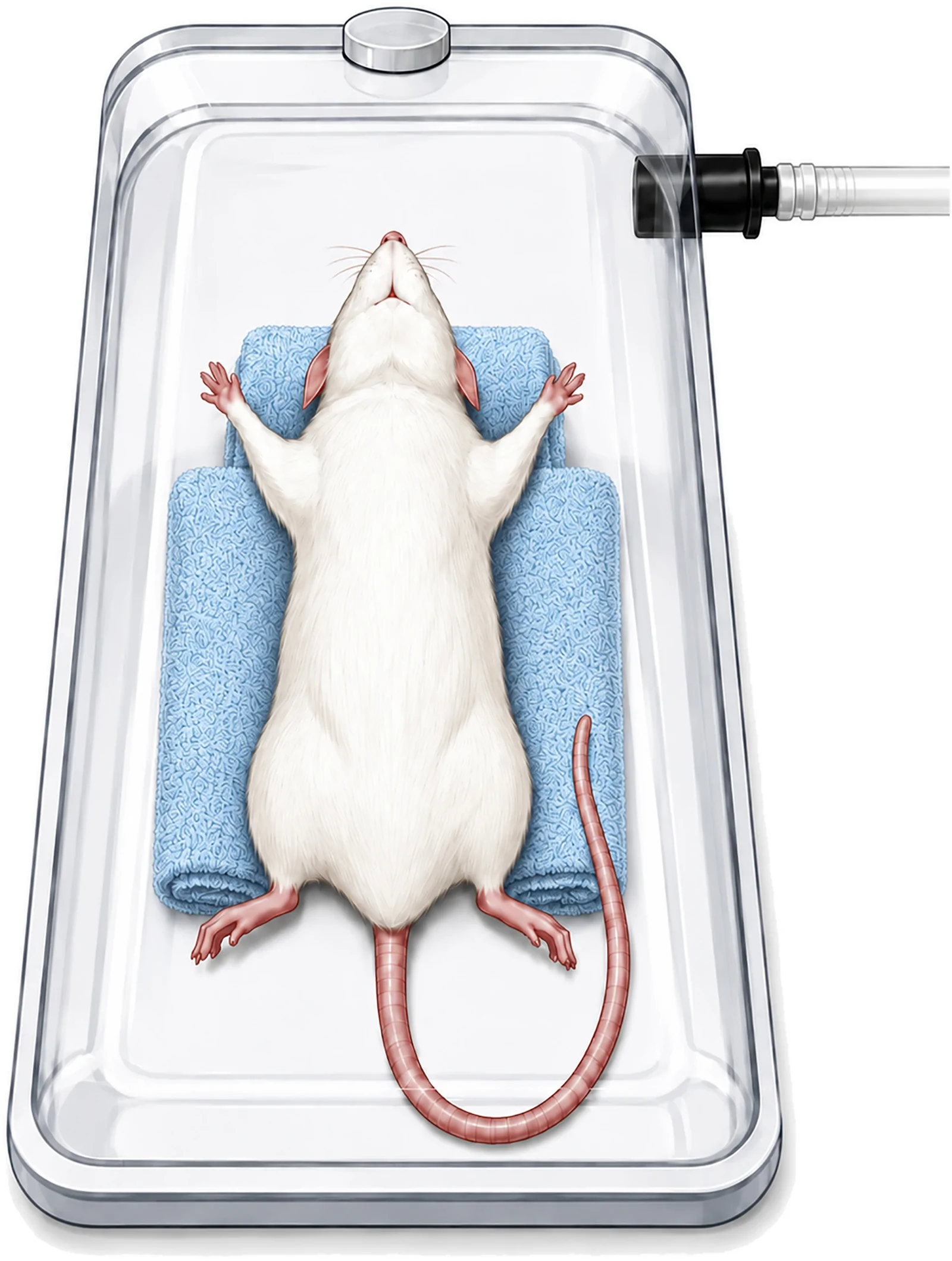
Postnasal infusion stabilization position Illustration showing a rat maintained in a dorsal supine position inside the induction chamber during the 15-min post-infusion stabilization period. Rolled towels support the animal laterally to maintain stable positioning and reduce loss of intranasally administered solution.

### Behavioral assessment of olfactory function

**Timing: 21 days (for step 7a)**

**Timing: Up to 3 weeks; up to 15 min per animal per test (for step 7b)**

The following steps are optional but recommended if users want to verify the presence, duration, and recovery of hyposmia-like olfactory impairment behaviorally. Olfactory function can be quantified using a hidden cookie test (modified buried food assay) performed at baseline and at defined time points following intranasal administration of gadolinium chloride or saline.

7. Hidden cookie test.
  a. Training (before nasal infusion).
    ***Note:*** Before intranasal gadolinium chloride or vehicle administration, rats should be trained to locate a chocolate-flavored cereal reward in the hidden cookie test. Training familiarizes animals with the testing environment and establishes stable olfactory-guided food-seeking behavior before induction of olfactory impairment.
    i. House rats in groups of 3–5 per home cage and, for at least 7 consecutive days before formal training, provide approximately two pieces of the cereal reward per rat in the home cage overnight. Confirm that animals readily approach and consume the reward.
    ii. Food-restrict animals for 16 h before each training session while maintaining ad libitum access to water. Conduct training during the light phase, typically in the morning.
    iii. Prepare a standard testing cage (59.5 × 38 × 20 cm) containing 3 cm of fresh sawdust bedding. Use Chocapic® chocolate-flavored cereal as the food reward throughout training and testing.
    iv. Gradually introduce the hidden-food task over 14 days: place five cereal pieces on the bedding on days 1–3; place two on the surface and bury three under 3 cm of bedding on days 4–6; bury all five under 3 cm of bedding on days 7–9; and bury one cereal piece under 3 cm of bedding on days 10–14.
    v. At the start of each trial, place the animal in the center of the cage facing forward and allow up to 15 min to locate and consume the cereal reward(s). Remove the animal immediately after retrieval; if the animal fails to retrieve the reward(s) within 15 min, end the trial and return the animal to its home cage.
    vi. Replace the bedding and clean the cage surfaces with 70% ethanol between animals and sessions. Allow the ethanol to evaporate fully before introducing the next animal.
    vii. Proceed to intranasal gadolinium chloride or vehicle administration only after animals reliably locate the single buried cereal piece within the predefined baseline criterion across consecutive trials.
  b. Testing phase after nasal infusion.
    ***Note:*** Testing assesses olfactory-guided food-seeking behavior after intranasal infusion. Testing should be performed after animals have completed training and reached stable baseline performance.
    i. Food-restrict animals for 16 h before testing while maintaining ad libitum access to water. Conduct testing in the morning.
    ii. Prepare the testing cage with 3 cm of fresh sawdust bedding and bury one piece of Chocapic® cereal under the bedding. Randomize the cereal location between testing sessions, while keeping the location consistent across animals within the same session.
    iii. Place the animal in the center of the cage facing forward and start the timer immediately. Allow up to 15 min to locate the cereal piece and record the latency to retrieval, defined as the time from placement in the cage until the animal fully exposes and grasps the cereal piece with its forepaws and/or mouth. Once the animal locates and retrieves the cereal, allow it to fully consume the reward before removing it from the testing cage.
    iv. If the animal does not locate the cereal within 15 min, end the trial and assign a latency of 15 min (900 s).
    v. Replace the bedding and clean the cage surfaces with 70% ethanol between animals and testing sessions. Allow the ethanol to evaporate fully before introducing the next animal.
    vi. Perform testing at baseline before intranasal administration and at defined post-infusion time points. Test the same animals repeatedly where appropriate and have latency scoring performed by an experimenter blinded to treatment group. Rats that have successfully mastered the hidden cookie task should reliably locate the buried reward in well under 1 min at baseline.
      **CRITICAL:** Increased latency should be interpreted as impaired olfactory-guided food-seeking behavior rather than direct evidence of complete anosmia. Visible food approach and locomotor activity controls are recommended to exclude major changes in motivation or gross motor function.
      ***Note:*** Testing frequency can be adapted to the experimental design. In our experiments, animals were typically tested every 2–3 days during the recovery period to monitor the onset, peak, and resolution of olfactory impairment. Hyposmia-like olfactory impairment typically lasts 7–10 days after intranasal gadolinium chloride administration.

### Data analysis

Latency to retrieve the cereal reward was analyzed as repeated-measures data, with treatment and time as factors. Animals that failed to locate the cereal reward within 15 min were assigned the maximum latency of 900 s. Because ceiling-level values may occur during peak impairment, data distribution should be assessed before selecting parametric or non-parametric tests. The same statistical approach should be applied to all animals before treatment allocation is unblinded.

## Expected outcomes

Intranasal administration of gadolinium chloride produces a robust and reversible impairment in olfactory-guided food-seeking behavior in adult male Wistar rats, as assessed using the hidden cookie test.

Under baseline conditions, prior to intranasal infusion, saline and gadolinium-treated animals reliably locate the hidden cookie with comparable latencies (28.1 ± 9.3s and 22.8 ± 10.7s, respectively), and no between-group differences are observed.

Following intranasal gadolinium administration, animals exhibit a marked and rapid increase in latency to locate the hidden cookie. The impairment is detectable within 24 h post-infusion and is most pronounced at day 3, during which a substantial proportion (10 of 11 rats; 91%) fail to locate the cookie within the 15 min testing window. In contrast, saline-treated animals maintain stable performance across all time points.

Behavioral impairment occurs on the first day following intranasal gadolinium, with recovery from approximately days 5–7 (Figure 3). By days 7–10 post-infusion, group-level performance is no longer significantly different from controls, and return to baseline olfactory-guided behavior is typically observed by approximately 2 weeks post-infusion.

**Figure 3:**
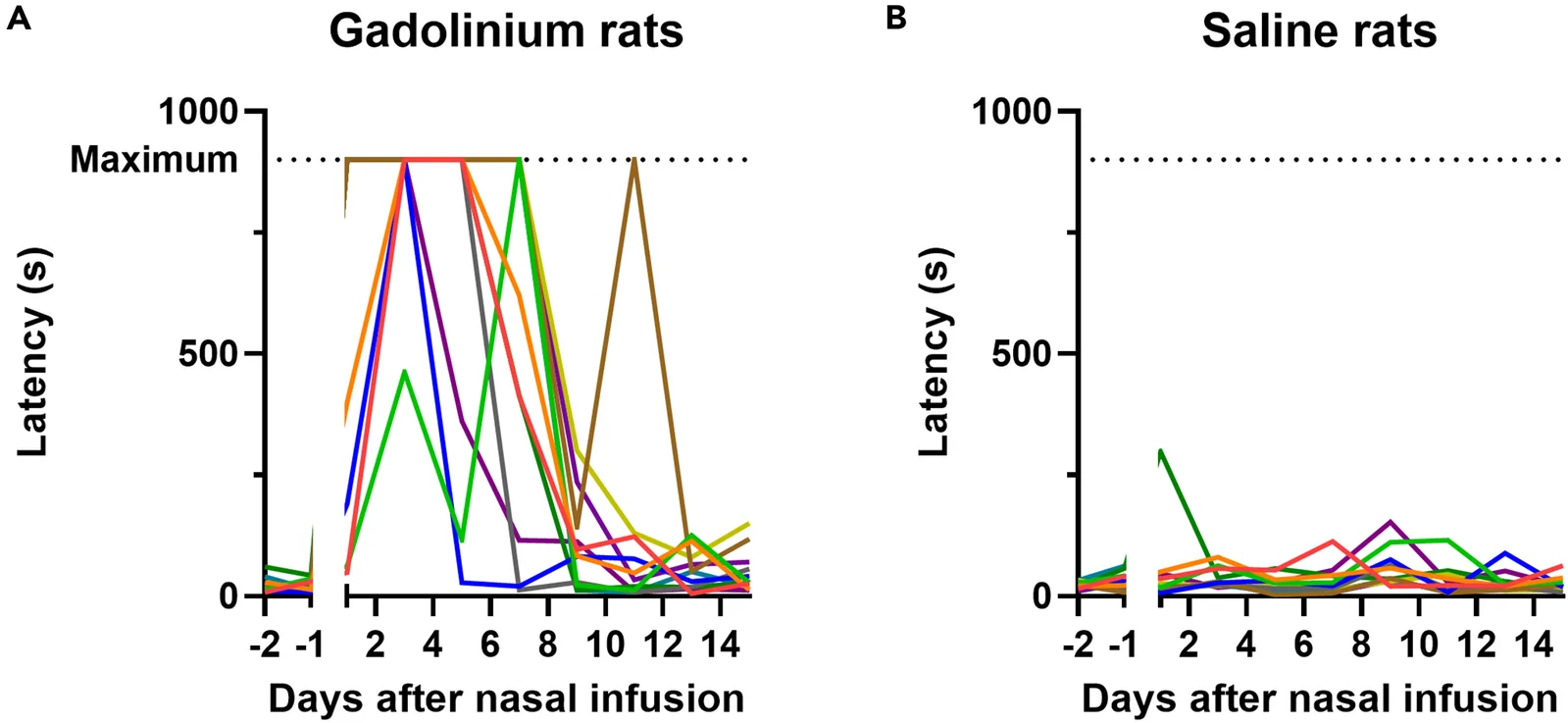
Hidden cookie test Time course showing changes in latency to find the hidden cookie 2 days before (−1, and −2) and after bilateral infusion of gadolinium (A) or saline (B) (50 microliter/nares) (N = 11 rats/group). Note the maximum time permitted for a rat to find the hidden cookie was 900 seconds. The colored lines represent different rats from both groups.

Performance in non-olfactory control measures, including approach to a visible food reward and locomotor activity in an open field, is unchanged following gadolinium administration,^4^ indicating that the observed deficit is not attributable to alterations in motivation or gross motor function.

Histological analyses performed in separate cohorts indicate that behavioral impairment coincides with transient disruption of the olfactory epithelium, including epithelial thinning and partial detachment from the basement membrane, most prominently at approximately day 5 post-infusion.^4^ These structural changes resolve by days 15–22, consistent with the reversible nature of the behavioral phenotype.

Transient disruptions in olfactory bulb electrophysiological activity have also been observed in separate cohorts, consistent with functional consequences of peripheral olfactory epithelium disruption.

A transient “snorting” sound may be observed during the first days following infusion, coinciding with acute nasal epithelial disruption, without evidence of respiratory distress or systemic illness. On the day of infusion, gadolinium chloride-treated animals may appear transiently lethargic and may show reduced interest in food or social interaction. These signs should resolve with recovery from anesthesia and should be distinguished from persistent distress, respiratory compromise, or systemic illness.

***Note:*** The larynx and pharynx were not assessed histologically in this study; therefore, potential microscopic irritation or injury to these regions cannot be excluded.

Overall, successful implementation of this protocol is indicated by the following temporal profile: normal baseline hidden cookie performance prior to infusion (20–30 s latency); marked impairment within 24 h; peak deficit between days 1–5 (frequent ceiling-level latencies of 15 min); recovery beginning around day 7; return to baseline olfactory-guided behavior by approximately 2 weeks post-infusion.

## Limitations

This protocol produces a transient impairment in olfactory-guided behavior consistent with hyposmia in adult male Wistar rats; however, the extent to which complete anosmia is induced has not been fully established. The behavioral phenotype has been primarily validated using the hidden cookie test, and sensitivity across alternative olfactory assays (e.g., odor discrimination or detection threshold tasks) has not yet been systematically assessed.

Additionally, interpretation of olfactory performance is dependent on a food-motivated behavioral assay. Although control experiments indicate no changes in motivation or locomotor activity following gadolinium administration, subtle behavioral confounds related to feeding motivation cannot be entirely excluded.

The efficacy and safety profile of intranasal gadolinium chloride delivery may vary depending on animal weight, anesthetic depth, and procedural technique. During the initial development of this protocol, respiratory complications were observed in a small number of animals when deeper anesthesia and smaller rats were used. Following optimization of the procedure—including the use of lighter anesthesia and larger adult rats (≥320 g)—no further respiratory complications were observed. Because these variables were refined simultaneously, the individual contribution of body weight cannot be determined; however, we recommend following the optimized protocol described here.

Finally, while transient histological disruption of the olfactory epithelium and changes in olfactory bulb electrophysiological activity have been observed in separate cohorts, these effects were not quantified in all behavioral experiments and should be interpreted as supportive rather than definitive mechanistic evidence.

## Troubleshooting

### Problem 1

Rat regains reflexes before infusion is completed.

### Potential solution

This usually indicates insufficient anesthetic depth. Return the animal to the induction chamber and extend anesthesia for 3–4 min before reattempting infusion. Proceed only once an appropriate anesthetic depth has been re-established, defined by absence of toe pinch reflex, absence of whisker movement, and regular spontaneous respiration.

### Problem 2

Infused solution is expelled from the naris.

### Potential solution

This may occur due to reflexive snorting, premature recovery from anesthesia, or incorrect head positioning. Ensure that the animal remains adequately anesthetized and that the head is maintained in a neutral, flat position during handling. If substantial solution loss occurs, record the event and exclude the animal according to predefined criteria. Do not administer additional gadolinium chloride unless repeat dosing has been specifically approved in the animal protocol.

### Problem 3

Respiratory distress occurs following bilateral infusion.

### Potential solution

Respiratory distress may result from partial obstruction of nasal airflow by the infused solution, particularly in smaller animals. Immediately discontinue anesthesia and reposition the animal in a prone position to restore airway patency and allow spontaneous airway control to recover. Monitor the animal continuously until normal respiration resumes. Avoid performing the procedure in rats weighing <300 g; animals weighing ≥320 g are recommended to reduce the risk of respiratory complications.

### Problem 4

Rat appears lethargic on the day of infusion.

### Potential solution

Mild transient lethargy may occur on the day of gadolinium chloride administration. Monitor respiration, posture, grooming, food and water intake, and social interaction. If lethargy persists beyond the expected post-anesthesia period or is accompanied by respiratory distress, cyanosis, failure to eat or drink, or abnormal posture, evaluate the animal according to local humane endpoint criteria and consult veterinary staff. In our experience, based on home cage activity, gadolinium chloride and saline rats are largely indistinguishable the following day.

***Note:*** Chocapic® chocolate-flavored cereal, Nestlé — one individual cereal piece was used as the hidden food reward. Equivalent dry, highly palatable cereal products may be used, provided that the same product and quantity are used consistently across experimental groups and testing sessions. Any alternative should be validated to ensure that it produces reliable odor-guided performance.

**CRITICAL:** The same reagents, equipment, and experimental conditions should be used across all experimental groups within a study. Equivalent equipment models and reagent brands may be used provided that they produce comparable performance and do not alter the procedure or outcome measures. Any substitutions should be validated before use. In particular, bedding depth, cookie type, cage dimensions, and other environmental conditions should be kept consistent because variability in these factors may affect latency measurements in the hidden cookie test.

## Resource availability

### Lead contact

Requests for further information, resources, and analyses should be directed to and will be fulfilled by the lead contact, Mark Jeremy Hunt (m.hunt@nencki.edu.pl).

### Technical contact

Technical questions on executing this protocol should be directed to and will be answered by the technical contact, Wiktoria Podolecka (w.podolecka@nencki.edu.pl).

### Materials availability

This study did not generate new unique reagents.

### Data and code availability

All data reported in this article will be shared by the lead contact upon request. This study does not report original code.

## Acknowledgments

This work was financed by the National Science Centre (Poland) OPUS 2021/41/B/NZ4/03882.

## Author contributions

W.P. carried out the experimental work. W.P. and M.J.H. analyzed the data. W.P. and M.J.H. wrote the manuscript.

## Declaration of interests

The authors declare no competing interests.
